# Integrating the milk microbiome signatures in mastitis: milk-omics and functional implications

**DOI:** 10.1007/s11274-024-04242-1

**Published:** 2025-01-18

**Authors:** Rine Christopher Reuben, Carmen Torres

**Affiliations:** 1 Biology Department, King’s College, 133 North River Street, Wilkes‐Barre, PA 18711 USA; 2 Area of Biochemistry and Molecular Biology, OneHealth-UR Research Group, University of La Rioja, 26006 Logroño, Spain

**Keywords:** Health, Mastitis, Milk, Microbiome, Omics

## Abstract

Mammalian milk contains a variety of complex bioactive and nutritional components and microorganisms. These microorganisms have diverse compositions and functional roles that impact host health and disease pathophysiology, especially mastitis. The advent and use of high throughput omics technologies, including metagenomics, metatranscriptomics, metaproteomics, metametabolomics, as well as culturomics in milk microbiome studies suggest strong relationships between host phenotype and milk microbiome signatures in mastitis. While single omics studies have undoubtedly contributed to our current understanding of milk microbiome and mastitis, they often provide limited information, targeting only a single biological viewpoint which is insufficient to provide system-wide information necessary for elucidating the biological footprints and molecular mechanisms driving mastitis and milk microbiome dysbiosis. Therefore, integrating a multi-omics approach in milk microbiome research could generate new knowledge, improve the current understanding of the functional and structural signatures of the milk ecosystem, and provide insights for sustainable mastitis control and microbiome management.

## Introduction

Mastitis is a frequently occurring polyetiological disease in dairy animals and lactating women. It is generally defined as a localized intramammary infection that often results in the inflammation of the mammary gland (Ali et al. 2021; Hu et al. 2022; Ruegg 2022; Chen et al. 2023a; Zhu et al. 2024a). Globally, mastitis is recognized in the dairy industry as the most devastating and major disease, causing significant economic losses and adversely impacting animal health and productivity as well as milk quality, safety, and yield (Silva et al. 2013; Hoque et al. 2019; Wang et al. 2022a; Ruegg 2022; Alessandri et al. 2023a; Panchal et al. 2024; Kizil et al. 2024). Depending on the severity of clinical manifestation and symptoms, mastitis presents either as a clinical or subclinical infection. While both subclinical mastitis (SCM) and clinical mastitis (CM) are characterized by high milk somatic cell counts (Song et al. 2020; Abed et al. 2021), the former however originates with varying conspicuous physiological and anatomical alterations, including irritation, inflammation, redness, and swelling of the mammary gland, resulting in changes in milk consistency, color, and yield. SCM usually presents as an inconspicuous infection with limited visible clinical symptoms yet causes significant impacts on the overall lactation performance, host health, and immune functioning (Kaczorowski et al. 2022; Wang et al. 2022b; Chen et al. 2023a). Due to the limited clinical signs, prolonged latency, and insufficient attention for prompt interventions, the incidence of SCM is substantially higher than CM, generally accounting for considerable (approximately 90%) cases of bovine mastitis (Song et al. 2020; Wang et al. 2022a). In humans, mastitis is estimated to occur in approximately 10 to 33% of all lactating women, though the reported incidence varies across geographical locations and populations (Pevzner and Dahan 2020; Wilson et al. 2020). While SCM in breastfeeding women is usually self-limiting and can resolve through self-management (e.g. breast massaging, cold compresses, etc.), CM requires treatment with antibiotics and cessation of breastfeeding in severe cases (Boix-Amorós et al. 2020; Wilson et al. 2020; Ouedraogo et al. 2022; Kizil et al. 2024).

Mammalian milk is often referred to as the “elixir of life” or “maternal white blood” readily available for consumption by offspring from birth. Being the sole nutrition for offspring from birth, milk is rich in its nutritional composition, essentially consisting of biologically active substances such as growth factors, oligosaccharides, and immunoglobulins as well as essential nutrients, including minerals, proteins, amino acids, vitamins, fats, water, etc. (Williams et al. 2017; Bruckmaier and Zinn 2023; Guo et al. 2024). Historically, milk was generally considered to be sterile unless contaminated either by external sources or due to [systemic] maternal infection (Fernández et al. 2020; Couvillion et al. 2023). From a traditional viewpoint, the presence of microorganisms in milk often indicates milk spoilage, mastitis, or a potential threat of the transmission of zoonotic or environmental pathogens to humans (Jones 1918; Holsinger et al. 1997; Fernández et al. 2013). Within the last decades however, numerous studies demonstrated the presence of microorganisms, especially lactic acid bacteria in milk from healthy hosts (Heikkilä and Saris 2003; Martín et al. 2003; Jiménez et al. 2015; Zimmermann et al. 2017; Togo et al. 2019b; Reuben et al. 2020; Zheng et al. 2020; Dehghani Champiri et al. 2023; Navarré et al. 2024; Akinyemi et al. 2024).

The increasing use of culture-independent high-throughput sequencing techniques has revealed the existence of diverse microbial communities across a wide range of niches, including body sites and fluids (e.g., milk) which were previously believed to be sterile when healthy (Zeineldin et al. 2017; Borghi et al. 2019; Oikonomou et al. 2020). Beyond the nutritional benefits derived from milk consumption, recent research continues to demonstrate the ubiquitous presence of highly diverse and previously unknown bacterial groups in milk (Murphy et al. 2017; Moossavi et al. 2019b; Toquet et al. 2021; Notarbartolo et al. 2022; Wang et al. 2023; Singh et al. 2023; Alessandri et al. 2023a). In comparison with other host and disease-associated microbiomes such as gut, skin, vagina, and respiratory tract, the milk microbiome and its relationship with mastitis are seldom studied together. The increasing understanding of niche-specific host-associated microbiomes and their impact on health has propelled interest in studying the milk microbial community as well as its impact on the health of both adults and offspring. Therefore, unraveling the relationship between milk microbiome and host health can present interesting and novel frontiers for improving infant and maternal health.

Through high throughput technologies, it is now possible to profile milk microbial communities and also elucidate their complex metabolic activity, functional potential, and host-microbe interactions. This could undoubtedly provide useful insights and increase current understanding of the relationship between any deviation in host health (e.g., in the case of mastitis) and the diversity and composition of milk microbial community and mediated metabolites. The milk microbiota and metabolites have been recently demonstrated to be the major determinant of milk quality, udder health status, and incidence of mastitis (Wang et al. 2020; Porcellato et al. 2020; Winther et al. 2022; Tarrah et al. 2022; Neculai-Valeanu and Ariton 2022; Alessandri et al. 2023a; Jin et al. 2023). Furthermore, recent studies have shown significant differences in the diversity and composition of milk microbial community and metabolites between CM, SCM, and healthy hosts (Wang et al. 2020, 2022a). Although the milk microbiome is yet to be extensively studied, there is a growing interest in understanding the extent of its dysbiosis in relation to the initiation and progression of mastitis in animals and humans. This review therefore provides significant insight into the possible drivers and sources of milk microbiome and their potential roles in mastitis and milk quality. We also discuss the advantages and challenges of different high throughput “omics” technologies, including metagenomics, metabolomics, metatranscriptomics, lipidomics, and metaproteomics separately and in combination (multi-omics) in elucidating the mechanistic relationship between milk microbiome and mastitis. The information provided will inform future experimental microbiome research and enhance the integration of functional and mechanistic microbiome potential in health and disease.

## Origin of milk microbial community: the entero-mammary, rumen-mammary, skin-milk, and retrograde hypotheses

The current ability to characterize the microbial community of milk and to unravel its origins has significantly expanded within the last two decades. This is largely due to the advancement of high throughput omics technologies in profiling microbial communities. Milk microbiota has been hypothesized to originate from both exogenous and endogenous sources, including mammary glands and entero-mammary microbial translation (Addis et al. 2016; Doyle et al. 2017; Moossavi et al. 2019a; Williams et al. 2019; Taponen et al. 2019; Power et al. 2024; Dombrowska-Pali et al. 2024). It has been traditionally believed that the milk microbiota originates exogenously from the surrounding environment, especially the skin of the mammary gland, teat canal, or the cavity of the offspring. However, recent advances in milk microbiota profiling reveal taxonomic groups that could not have originated from exogenous sources (Gueimonde et al. 2007; Jost et al. 2014; Pannaraj et al. 2018; Moossavi and Azad 2020; Filatava et al. 2023; Power et al. 2024; Guo et al. 2024; Dombrowska-Pali et al. 2024). Therefore, the external or surrounding environment cannot be solely considered as the source of milk microbiota.

The evidence of the endogenous origin of milk microbiota has been supported by different studies involving humans, mice, and ruminants (Perez et al. 2007; Jiménez et al. 2008; Young et al. 2015; de Andrés et al. 2017; Ma et al. 2018; Hoque et al. 2022; Xu et al. 2024b). The endogenous origin of milk microbiota through entero-mammary translocation of microorganisms has garnered considerable attention over the years. This is because microbial communities across ecological niches within the host do not function independently as separate environments. They closely interconnect and interact with each other, forming a network of complex inter-related microbial communities. Consequently, microorganisms from the gut and other body sites may enter the mammary gland and eventually the milk through endogenous routes (Costello et al. 2009; Ruegg 2022; Guo et al. 2024). The existence of entero-mammary pathways and the transfer of microorganisms from the gastrointestinal tract to the mammary glands have been described by several authors (Costello et al. 2009; Donnet-Hughes et al. 2010; Fernández et al. 2013; Jost et al. 2014; Stinson et al. 2021).

Though not clearly elucidated, the mechanisms regulating microbial translocation across the intestinal barrier to the mammary glands or milk are widely believed to selectively involve immune cells, especially macrophages and intestinal dendritic cells (Martín et al. 2004; Perez et al. 2007; Rodríguez et al. 2021; Selvamani et al. 2021; Guo et al. 2024; Dombrowska-Pali et al. 2024). The intestinal dendritic cells selectively sample gut contents by loosening the tight junctions between intestinal absorptive cells and extending their dendrites to the lumen without compromising the barrier integrity of the intestinal epithelia (Rescigno et al. 2001; Donnet-Hughes et al. 2010; Rodríguez et al. 2021). Because of the sampling activity, the dendritic cells can selectively harbor and transfer live bacteria to the mesenteric lymph nodes which consequentially spread to the lactating mammary gland and other distant mucosal surfaces through the lymphoid system of the mucosa (Donnet-Hughes et al. 2010; Ferretti et al. 2018; Ruegg 2022). More so, active lactation causes the migration of cells from the intestinal lymphoid tissues to the mammary gland through peripheral and lymphatic blood circulations (Ruegg 2022). The presence of bacteria and their genetic material have been previously reported in human peripheral blood mononuclear cells and breast milk cells during lactation (Rodríguez 2014; Ferretti et al. 2018; Rodríguez et al. 2021).

For instance, strains of lactic acid bacteria orally administered to mice and rats during pregnancy and lactation were equally detected in milk and mammary tissues of the treatment group but not in the untreated group (control) (de Andrés et al. 2017; Azagra-Boronat et al. 2020b, a; Selvamani et al. 2021). Similarly, *Lactobacillus gasseri* CECT5714, *Ligilactobacillus salivarius* CECT5713, and *Limosilactobacillus fermentum* CECT5716 were detected in breast milk of lactating mothers following oral consumption of specific probiotics containing the same microbial strains (Derakhshani et al. 2018; Stinson et al. 2021). Although further research is required to fully unravel the underlying mechanisms of entero-mammary translocation of microorganisms, however, these findings suggest strong evidence of the transfer of microorganisms from the gut to the mammary glands and eventually milk.

In support of the endogenous origin of milk microbiota, additional studies (Young et al. 2015; Metzger et al. 2018; Jiang et al. 2023) have detected rumen microbiota and genetic material in bovine milk, hence suggesting the rumen-mammary pathway in ruminants. Similarly, bovine milk shares a physiological resemblance with rumen contents in terms of physicochemical composition (e.g. temperature and pH) and rich nutritional composition that support microbial growth (Priyashantha et al. 2021; Souza et al. 2022; Guo et al. 2024). Interestingly, these compelling similarities plausibly support the interconnectedness and crosstalk between the microbiota of rumen and bovine milk. The detection of certain rumen anaerobic microorganisms, including *Bifidobacterium* spp, *Ruminococcus* spp, and members of Peptostreptococcaceae family in bovine milk of healthy lactating cows (Young et al. 2015; Jiang et al. 2023) further supports the rumen-mammary hypothesis. The presence of these obligate anaerobic microorganisms in bovine milk suggests that the surrounding environment and exogenous sources cannot be considered the sole origin of milk microbiota (Lima et al. 2017; Taponen et al. 2019).

The retrograde flow of infant oral microorganisms into the breast and mammary ducts of lactating mothers as documented in human studies (Murphy et al. 2017; Ferretti et al. 2018; Moossavi et al. 2019b; Williams et al. 2019; Fehr et al. 2020; Ames et al. 2023) has been hypothesized as another likely source of maternal milk microbiota. The microbiome composition and structure of infants’ oral cavities are notably similar to that of maternal milk (Biagi et al. 2017; Avershina et al. 2018; Williams et al. 2019; Couvillion et al. 2023). Supporting this hypothesis, several studies have reported the similarity (especially the dominance of *Streptococcus* spp) between the maternal milk microbiome and that of the infant oral cavity (Hunt et al. 2011; Cephas et al. 2011; Fernández et al. 2013; Williams et al. 2019; Nardi et al. 2021; Arishi et al. 2023). The infant oral microbiota has been estimated to contribute to about 21% and 66% of maternal milk microbiota at day 2 and 5 months of age (Williams et al. 2019). Undoubtedly also, the maternal milk microbiome plays a vital role in colonizing and shaping the microbiome of the infants’ oral cavity (Williams et al. 2019; Ruiz et al. 2019a). The retrograde pathway is rarely documented in dairy cows and other animals. This is partly because most dairy farms often restrict the interactions between the cows and their calves shortly after birth, thereby limiting microbial interactions through the suckling of maternal milk. In addition to the retrograde flow of microorganisms from the infant oral cavity to the mammary ducts, breast skin microbiota may also contribute to maternal milk microbial composition. Milk contains species of *Staphylococcus, Corynebacterium*, and *Propionibacterium* which are notable inhabitants of adult skin including the sebaceous breast skin (Latuga et al. 2014; Oh et al. 2014; Jiménez et al. 2015; Oikonomou et al. 2020; Nardi et al. 2021).

## Core milk microbiota in healthy hosts: human, bovine, and small ruminants

### Human milk microbiota

The human milk microbiota (HMM) is highly diverse and complex, consisting of over 800 species of bacteria with the majority being obligate aerobic or facultative anaerobic bacteria (Togo et al. 2019a; Lyons et al. 2020; Notarbartolo et al. 2022; Ajeeb et al. 2024; Power et al. 2024; Dombrowska-Pali et al. 2024). The presence of these anaerobic bacteria in HMM is known to beneficially impact infants’ health and well-being (Lyons et al. 2020; Kashyap and Choudhari 2024; Dombrowska-Pali et al. 2024). Over the years, several studies have characterized the HMM using both the culture-dependent and culture-independent approaches with the latter widely used in recent years. In a systematic analysis comprising 15,489 milk samples from 11,124 women across 38 countries, 820 bacterial species that belonged to 178 genera, 92 families, 52 orders, 24 classes, and 13 phyla were identified from human milk (Togo et al. 2019a). While some phyla (e.g. Fusobacteria, Deferribacterota, Cyanobacteria) have relatively lower abundance, commonly identified genera in milk from healthy women include *Staphylococcus*, *Streptococcus*, *Corynebacterium*, *Pseudomonas*, *Serratia*, *Propionibacterium*, *Bradyrhizobium, Sphingomonas, Ralstonia*, *Cutibacterium*, *Enterococcus*, *Lacticaseibacillus*, *Lactiplantibacillus, Limosilactobacillus, Lactococcus*, *Lactobacillus*, *Leuconostoc*, *Bifidobacterium*, and *Weissella* and other taxonomically-related Gram-positive bacteria (Togo et al. 2019a; Fernández et al. 2020; Ajeeb et al. 2024). Although previous studies involving multiple countries demonstrated varied HMM across geographical locations, however, consistent and universal members of the HMM were identified as the core genera in all the samples analyzed (Fitzstevens et al. 2017; Lackey et al. 2019).

Increasing reports have demonstrated the HMM to contain organized consortia and networks of bacteria that are often stable in structure, diversity, and abundance throughout the lactation period (Sam Ma et al. 2015; Drago et al. 2017; Fernández et al. 2020; Holdsworth et al. 2023). Regardless of the maternal body mass index (BMI), health, diet, demographics, and geography, four dominant phyla including Bacteroidetes, Proteobacteria, Firmicutes, and Actinobacteria are usually identified across human milk samples (Togo et al. 2019a; Fernández et al. 2020; Notarbartolo et al. 2022; Banić et al. 2022; Dinleyici et al. 2024; Wang et al. 2024a; Ajeeb et al. 2024). Among these dominant phyla, previous research has documented a nine-genera core bacteria that constitute HMM, comprising *Staphylococcus*, *Streptococcus*, *Corynebacterium*, *Pseudomonas*, *Serratia*, *Propionibacterium*, *Bradyrhizobium, Sphingomonas* and *Ralstonia* (Demmelmair et al. 2020; Moubareck 2021; Notarbartolo et al. 2022; Wang et al. 2023; Dinleyici et al. 2024; Dombrowska-Pali et al. 2024). Interestingly, the core bacteria in human milk represent about half of the HMM. However, their relative abundance appears to be variable among milk samples, geography, experimental techniques, and analysis (Diez-Sampedro et al. 2019; Moubareck 2021; Cheema et al. 2023). In addition, potential mother-to-infant microbial transmission through breastfeeding, as is the case with *S. aureus,* which can also colonize the infant intestine, has been previously shown (Benito et al. 2015).

### Bovine milk microbiota

The last two decades have witnessed a substantial increase in research exploring the entire bovine milk microbiota (BVM) rather than specific milk-borne pathogens. Several comparative studies have consistently reported the differences in the composition and structure of BVM in both healthy and diseased (mastitis) cows (Derakhshani et al. 2018; Hoque et al. 2019, 2020; Couvillion et al. 2023; Khasapane et al. 2023; Yang et al. 2024; Power et al. 2024; Guo et al. 2024; Salman et al. 2024). The complexity of the BVM shows that bovine milk contains a highly abundant, complex, and diverse microbial community. Similar to the HMM, the BVM harbors core phyla and genera that are considerably conserved and consistently appear in at least 95% of all bovine milk samples regardless of dietary, environmental, and individual variations in cows (Astudillo-García et al. 2017; Moossavi et al. 2019b; Ryu et al. 2021; Guo et al. 2024). While some studies have reported inconsistencies in the compositions of the BVM across individuals and geographical locations, others have shown relative stability in core microbial groups as well as their overall metabolic/physiological properties and functionalities (Mizrahi et al. 2021; Guo et al. 2024).

A recent study indicated the presence of 119 bacterial species from 202 genera, 124 families, 82 orders, 33 classes, and 95 phyla from 166 composite milk samples obtained from 166 individual dairy cattle in South Africa (Khasapane et al. 2023). Notably, four core-phyla including Proteobacteria, Firmicutes, Bacteroidota, and Actinobacteria were present in over 97% of the total samples evaluated. In a previous study comprising 112 milk samples from individual cows from 10 different farms in the Shanghai region of China, 33 phyla and 785 genera were detected (Li et al. 2018). The core bacterial groups identified included four phyla [Bacteroidetes (7.47%), Actinobacteria (9.40%), Proteobacteria (39.0%), and Firmicutes (40.8%)] and four genera [*Acinetobacter* (10.2%), *Lactococcus* (11.7%), *Bacillus* (13.8%), and *Pseudomonas* (19.6%)] in all the samples. Similarly, several recent studies separately revealed the presence of the four core-phyla from milk samples from dairy cattle in Ireland, Pakistan, Turkey, Japan, China, Italy, Bangladesh, Korea (Hoque et al. 2019; Ryu et al. 2021; Kizil et al. 2024; Yang et al. 2024; AoDaohu et al. 2024; Yap et al. 2024; Salman et al. 2024). The core BVM is generally believed to consist of *Bacteroides*, *Staphylococcus*, *Lactobacillus*, *Propionibacterium, Enterococcus*, *Streptococcus*, *Lactococcus*, *Porphyromonas*, *Corynebacterium*, *Fusobacterium,* and *Pseudomonas* (Addis et al. 2016; Hoque et al. 2019; Oikonomou et al. 2020; Porcellato et al. 2020; Power et al. 2024; Guo et al. 2024). Interestingly, some of these genera are often associated with healthier udder-quarters in cows (Addis et al. 2016).

### Small ruminants’ milk microbiota

The structure and composition of the milk microbiota of small ruminants are highly variable probably due to the limited number of studies as well as several environmental and individual species/breed-specific factors. Milk microbiota of small ruminants such as goat, sheep, reindeer, and water deer show significant differences, thus suggesting environmental influences, host-associated factors, and host-microbial adaptation as major drivers of microbial composition and structure (Li et al. 2017; Oikonomou et al. 2020; Polveiro et al. 2022; Guo et al. 2023; Hoving-Bolink et al. 2023). So far, there has been no consensus on the overall core microbial phyla or genera in the milk of small ruminants across various species or breeds. Out of 31 and 43 phyla identified from the milk samples of 212 Spanish Churra sheep breed and 50 Assaf ewes, Actinobacteria, Bacteroidetes, Firmicutes, and Proteobacteria accounted for 97.4% and 90.07% of all the samples examined (Esteban-Blanco et al. 2020b, a). While Actinobacteria, Firmicutes, and Proteobacteria were reported as the core phyla in the milk microbiota in sheep (Esteban-Blanco et al. 2020b, a), the presence of other recurring phyla, especially Bacteroidetes, Acidobacteria, Cyanobacteria, Fusobacteria, have been documented as well (Castro et al. 2019; Esteban-Blanco et al. 2020b, a). Although over 1000 genera were identified from sheep milk, studies have shown *Corynebacterium*, *Lactobacillus*, *Staphylococcus*, *Streptococcus,* and *Escherichia/Shigella* to be the core microbiota of milk from healthy sheep (Castro et al. 2019; Esteban-Blanco et al. 2020b, a; Toquet et al. 2021). However, host-related factors such as breed as well as geographic locations have been suggested to impact milk microbiota composition in sheep (Castro et al. 2019; Esteban-Blanco et al. 2020a).

Goat milk microbiota is reported to primarily contain Firmicutes and Proteobacteria as the core phyla and to a minor extent, Actinobacteria (Li et al. 2017; Zhang et al. 2017; Niyazbekova et al. 2020; Polveiro et al. 2022; Lauková et al. 2022; Hoving-Bolink et al. 2023). These phyla usually constitute about 90% of the total bacterial phyla in milk from healthy goats. Furthermore, studies have shown phylum-level variations in goat milk microbiota composition during the lactation period (McInnis et al. 2015; Zhang et al. 2017; Niyazbekova et al. 2020). In a recent study, Hoving-Bolink et al. (2023) identified *Lactococcus*, *Staphylococcus*, *Pseudomonas*, *Acinetobacteria*, *Corinebacteria*, and *Microbacteria* as the core genera in healthy goat milk. Polveiro et al. (2022) reported the presence of *Staphylococcus* spp, *Brevidabacterium* spp, *Enterococcus*, and *Bacteroides* spp in all the milk samples examined, including healthy goats and goats diagnosed with clinical, subclinical, and gangrenous mastitis. Whereas *Curtobacterium*, *Staphylococcus*, and *Bifidobacterium* were reported as the core genera in milk samples from healthy goats across farms in central and eastern Slovakia, the presence of *Enterococcus*, *Lactococcus*, *Streptococcus*, *Lacticaseibacillus*, and *Lactobacillus* was also prevalent (Lauková et al. 2022). Factors including animal breed, sample origin, farm location, and management appear as the key drivers in goat milk microbial composition and structure.

### Mastitis: dysbiosis of milk microbiota

Intramammary infections resulting in mastitis constitute a common disease in mammalian species globally. Mastitis usually causes a significant decrease in milk production, dysbiosis of the milk microbiota, undesired weaning, premature culling, difficulty in conception, and treatment costs in both humans and animals (Wolfenson et al. 2015; Boix-Amorós et al. 2020; Fernández et al. 2020; Wang et al. 2021a; Borş et al. 2023; Ito et al. 2023; Crippa et al. 2024). Mastitis affects approximately 10 to 33% of all lactating women resulting in severe public health problems in both infants and mothers (Pevzner and Dahan 2020; Wilson et al. 2020). In animals especially cattle, up to 15% decrease in milk production, overall animal well-being, and behavioral changes due to mastitis have been well-documented (Addis et al. 2016; Toquet et al. 2021; Morales-Ubaldo et al. 2023).

Mastitis is often characterized by the dysbiosis of the milk (and mammary) microbiota, depending on the clinical manifestations (clinical or subclinical) or the course (acute, granulomatous, and subacute) (Angelopoulou et al. 2018; Demmelmair et al. 2020; Fernández et al. 2020; Dobrut et al. 2024). The analysis of mastitis milk from humans and animals provides new insights into the extent of milk microbiota perturbations as well as the ecology of mastitis-associated etiologies. As previously mentioned, milk from healthy hosts contains highly diverse bacteria with the majority regarded as nonpathogenic and often unrelated to mastitis. The role of these bacterial groups in the initiation, progression, and prevention of mastitis is not fully elucidated. However, emerging evidence showed the potential impact of the milk microbiota in the development of mastitis (Hoque et al. 2022; Ito et al. 2023; Yang et al. 2024; AoDaohu et al. 2024; Guo et al. 2024; Salman et al. 2024). Milk from mastitis-suffering animals and women shows distinct microbiota composition and structure when compared to healthy hosts (Mediano et al. 2017; Hoque et al. 2019; Selma-Royo et al. 2021; Toquet et al. 2021; Kizil et al. 2024; Yang et al. 2024; Salman et al. 2024). The milk microbiota from acute and subacute mastitis is often distinct in both structure and composition with an abundant presence of aerotolerant bacteria, especially *Staphylococcus*, and significantly reduced diversity and beneficial obligate anaerobes, including *Faecalibacterium*, *Eubacterium, Ruminococcus*, etc. (Patel et al. 2017; Derakhshani et al. 2018; Esteban-Blanco et al. 2020b; Boix-Amorós et al. 2020). In a study consisting of 1849 milk samples from individual lactating women with mastitis (acute/subacute), 91.56% and 29.74% of the milk examined revealed the presence of *Staphylococcus epidermidis* and *Staphylococcus aureus* (Mediano et al. 2017). Additionally, streptococci (70.20%) and corynebacteria (16.60%) constituted the dominant microbial groups from the milk analyzed. The presence of *S. epidermidis* was previously reported in 85% of milk from women with mastitis (Delgado et al. 2008). While *Staphylococcus* is the core genus associated with acute and subacute mastitis, *S. aureus* and *S. epidermidis* are the common staphylococci frequently isolated from mastitis milk (Jiménez et al. 2015; Boix-Amorós et al. 2020). Although *Pseudomonas, Klebsiella, Serratia, Ralstonia, Aeromonas,* and *Enterococcus* are other enriched and frequently isolated genera from mastitis milk, *Clostridium, Ruminococcus, Faecalibacterium, Acinetobacter,* and *Eubacterium* are consistently depleted in milk samples of subacute and acute mastitis (Patel et al. 2017; Angelopoulou et al. 2018; Hoque et al. 2022). The lower microbial diversity characterizing mastitis milk microbiota consequentially increases the presence of opportunistic pathogens *Escherichia coli*, *Bacillus subtilis, B. cereus, E. faecalis, S. epidermidis, S. hominis,* and *K. pneumoniae.* In comparison to milk from healthy individuals, mastitis milk contains a significantly higher presence of *Staphylococcaceae, Brucellaceae, Burkholderiaceae, Streptococcaceae,* and *Aeromonadaceae* at the family level as well as a higher presence of *Staphylococcus, Streptococcus, Ralstonia, Klebsiella, Aeromonas, Leptospira,* and *Proteus* at the genus level (Boix-Amorós et al. 2020; Ito et al. 2023; Khasapane et al. 2023; Singh et al. 2023; Jin et al. 2023).

Bovine mastitis in cows is characterized by an increased presence of *Mycoplasma* spp, *Streptococcus dysgalactiae*, *Streptococcus agalactiae*, *Streptococcus uberis, S. aureus, E. coli, Klebsiella pneumoniae,* and *Corynebacterium bovis* in cow milk (Falentin et al. 2016; Belay et al. 2022; Girma and Tamir 2022; Morales-Ubaldo et al. 2023). Belay et al. (2022) identified *S. aureus* (42.6%), *Streptococcus* spp. (26.2%), non-*aureus* staphylococci (14.8%), *E. coli* (11.5%), *Salmonella* spp (3.3%), and *K. pneumoniae* (1.6%) as the predominant bacterial species in 422 milk samples of lactating cows diagnosed with mastitis. Whilst the most abundant genera of bacteria in mastitis cow milk were reported to include Bacilli, Clostridia, Alphaproteobacteria, Actinobacteria, Gammaproteobacteria, the dominant bacterial species include *Pseudomonas koreensis, P. azotoformans*, *P. fragi, Acinetobacter guillouiae*, and *Mycobacterium bovis* (Khasapane et al. 2023). In addition to the core microbial groups in bovine milk as previously mentioned, bovine mastitis results in the additional presence of enriched bacterial species including *Staphylococcus hominis*, *Lactobacillus acidipiscis,* and four unknown species of the genus *Tetragenococcus, Mogibacterium*, *Jeotgalicoccus, Hymenobacter*, *Lachnospiraceae,* and *Anaerococcus* (Alessandri et al. 2023a; Burakova et al. 2023). The decrease in the abundance of beneficial bacterial groups such as *Atopostipes, Massilia*, *Acetitomaculum*, *Ralstonia* in the milk from cows with mastitis demonstrates their role in the maintenance of eubiosis and healthy milk microbiota (Burakova et al. 2023). Interestingly, the milk microbiota of goats with mastitis are highly diverse and appear to be dominated by Fusobacterium, Bacteroides, and Proteobacteria when compared to milk from healthy goats (Polveiro et al. 2020; Toquet et al. 2021). Analysis of milk from ewes with mastitis revealed the same genera constituting the core microbiota (in healthy ewes) as mentioned above as well *as Clostridium* spp, *Turicibacter* spp, *Romboutsia* spp, *Jeotgalicoccus* spp, *Pseudomonas* spp, and *Alloicoccus* spp (Esteban-Blanco et al. 2020b). In the same vein also, milk from ewes previously known to suffer from mastitis had diverse bacterial species consisting of *Sphingobacterium spiritivorum, Staphylococcus warneri, S. schleiferi*, *S. equorum*, *S. haemolyticus, S. felis, Pseudomonas aeruginosa, Enterococcus hirae, Clavibacter michiganensis, Bacillus pumilus*, *Mannheimia haemolytica,* and *Corynebacterium* spp (Gelasakis et al. 2015; Castro et al. 2019). Recently, (Couvillion et al. 2023) suggested the existence of a causal relationship between host phenotypes with mastitis and milk microbiota.

### Characterization of milk microbiome

**Fig. 1:**
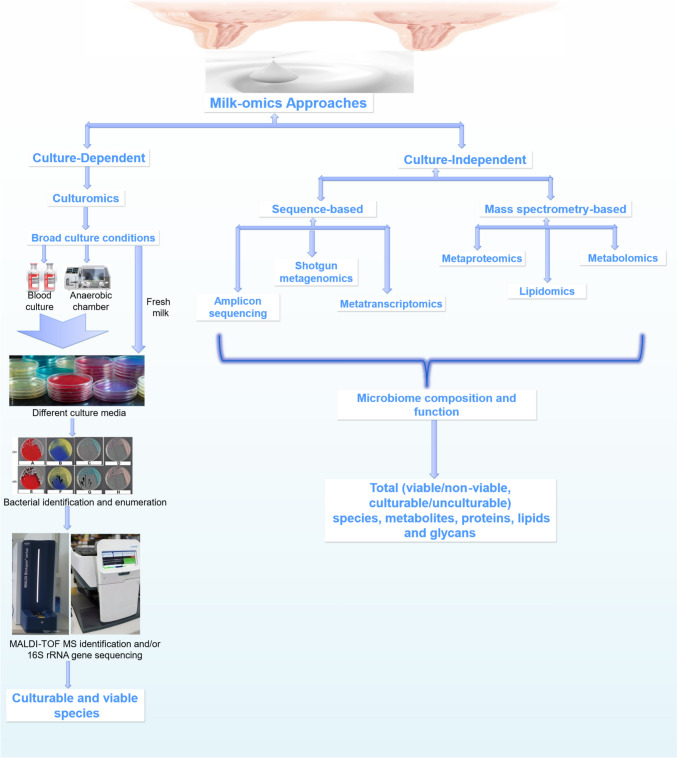
Experimental flow chart of milk-omics analyses

The experimental methodologies for profiling the milk microbiota continue to evolve. The advent of high throughput technologies for microbiota characterization in addition to the classic microbiological methods shows that the methods/techniques used in microbiota profiling are pivotal in uncovering the observed taxa or bacterial groups in milk (Lopez Leyva et al. 2021; Notarbartolo et al. 2022; Selma-Royo et al. 2022; Cheema et al. 2023). So far, the experimental methods for the analysis of milk microbiota rely on both the traditional culture-dependent techniques as well as culture-independent methods (Fig. 1) which primarily depend on the nucleic acids sequence-based approaches, including amplicon sequencing, shotgun metagenomics, and metatranscriptomics (Table 1).

**Table 1:** Features of milk-omics approaches available for studying microbial communities

Omics approach	Platform	Target molecule(s)	Analytical workflows	Applications	Advantages	Drawbacks
Amplicon sequencing	Sequencing-based	DNA	Marker (16S rRNA) gene next generation sequencing	Taxonomic distribution and profiling	Commonly used method with comprehensive reference databases Cost-effective and scalable Applicable to low-biomass	Characterizes only bacteria Primers and PCR biases Unable to provide functional information
Metagenomics	Sequencing-based	DNA	Next generation sequencing	Taxonomic profiling of microbial community composition and structure Microbiota characterization, Captures community gene composition Assessment of potential functional properties and microbial biomarkers	Recovery of whole genomes Greater taxonomic resolution, Overcomes amplification biases Reveals potential functional and metabolic properties	Cannot inform if genes are actively expressed Data complexity and sequence annotation issues High cost and computational requirements Contamination with host DNA
Metatranscriptomics	Sequencing-based	RNA	Next generation sequencing	Assessment of microbial activity Taxonomic distribution and gene expression Assessment of microbiome-environment interactions Analysis of phylogenomic profiles of microbiome	Assess the functionality of the microbiome Determine the association between gene expression and microbial phenotypes Provides insight into functionally active metabolic pathways, interactions with host/environment, and functional alterations	Issues with RNA extraction and stability Contamination with host RNA Issues with data analysis, optimization, and interpretation of uncharacterized transcripts Difficulty with data integration with other omics approach
Metaproteomics	Mass spectrometry-based	Proteins	Mass spectrometry	Determination of protein composition of microbial communities Assessment of community composition and species-level biomass contributions Assessment of interactions between community members Profiling of expressed community metabolism and physiology Determination of protein biomarkers Assess substrate utilization by specific community members	Provide taxonomic composition, distribution, and functionality of microbiomes Functional profiling through activity-based protein expressions Determination of interactions between community members Provides insights into gene expression in the health and disease of both microbes and host	Wide range and variability of proteins Issues with coverage and accuracy in detecting proteins from less abundant microbial species Lack of standardization and reproducibility, and sample preparation protocols Issues with high computational demand and data interpretation Lack of comprehensive and optimized protein sequence database
Metametabolomics	Mass spectrometry-based	Metabolites/organic compounds	Mass spectrometry	Assessment of microbial metabolites, host-microbe co-metabolism, and interactions, exogenous molecules (diets, xenobiotics, etc.) Identification of metabolites and biomarkers Profiling of metabolic fluxes Determination of dynamics of metabolism through flix balance analysis	Provides overall metabolic fluxes and the physiological state of the microbiome Assessment of regulation and dysregulation of metabolic pathways in health and disease	Difficulty in linking metabolite and microbial taxonomy Only a limited number of metabolites have been detected Challenges with the annotation of detected metabolites Lack of comprehensive reference databases and bioinformatics pipelines
Lipidomics	Mass spectrometry-based	Lipids	Mass spectrometry	Characterization and quantification of the structure and function of intact lipid molecules Analyses the interplay between the microbiota and lipids	Provides useful insights into bioactive lipids-mediated inflammatory responses and changes in lipid contents Provides valuable insights into disease diagnosis, Assess lipid-microbiota interactions in ecosystems	Lack of conventional standards for absolute lipid quantification at the species level Unavailability of a potent lipid separation system capable of differentiating phospholipids from triacylglycerols Lack of comprehensive reference databases and bioinformatics pipelines

### Culture-dependent approaches: milk culturomics

The initial microbiological studies on milk relied on traditional culture-dependent techniques to characterize milk microorganisms. The culture-based techniques assess the morphological, phenotypic, and biochemical characteristics of the isolated strains which are sometimes genotypically identified. Culture-based conditions are often biased toward identifying pathogens as well as viable and dominant bacteria. However, fastidious, non-culturable, and less occurring bacteria are usually not detected (Ruiz et al. 2019b; Lopez Leyva et al. 2021; Cheema et al. 2023). Though powerful in profiling the viability of specific milk-borne bacteria, culture-based techniques only reveal limited taxa capable of withstanding sampling procedures, transportation, storage, and experimental/laboratory conditions. Consequentially, these techniques could selectively reduce the depth of the overall microbial community, detecting only a minute diversity of the bacterial taxa in milk (Browne et al. 2016; Sakwinska and Bosco 2019; Cheema et al. 2023). For instance, from the 554 bacterial species identified in human milk, only 210 species (38%) were detected by culture-based techniques (Togo et al. 2019a). Through these techniques, the presence of dominant facultative anaerobes and pathogenic bacteria associated with mammary infections including *Streptococcus, Staphylococcus, Propionibacterium*, and *Corynebacterium* (Martín et al. 2003; Ruiz et al. 2019b). Additionally, bifidobacteria and several lactic acid bacteria, especially *Enterococcus*, *Weissella*, *Lactobacillus*, *Lactococcus, Leuconostoc*, etc. have been successfully detected in milk after using nutrient-specific culture media and regulated incubation conditions (Abrahamsson et al. 2009; Albesharat et al. 2011; Martín et al. 2011; Murphy et al. 2017; Breitenwieser et al. 2020; Selma-Royo et al. 2021; Damaceno et al. 2023; Wang et al. 2024a). Despite the limitations of the culture-based techniques, culturing facilitates the exploitation and preservation of bacterial strains for potential applications in biotechnological, health, and agrifood systems. Apart from the frequent milk microbiological studies involving the distribution of [pathogenic] bacteria as well as their antimicrobial resistance and virulence determinants, potentially beneficial traits such as probiotic properties, bacteriocin production, and other biotechnologically important potentials are extensively sought from milk-borne bacteria (Zhang et al. 2022c; Kim et al. 2022; Damaceno et al. 2023; Asha et al. 2024; da Cunha et al. 2024; Elnar and Kim 2024).

The recent decades have witnessed the advent and development of a culture-based microbiota approach known as culturomics. Culturomics is a highly effective culture-dependent technique that uses high-throughput and specific microbial culture conditions for large-scale isolation and rapid identification of bacteria in a community (Lagier et al. 2016, 2018; Ruiz et al. 2019b; Cheema et al. 2023). Culturomics approach facilitates the collection of a comprehensive repertoire of the microbiota and also the detection of species with low abundance which are often undetectable by culture-independent methods, including metagenomics (Seck et al. 2019; Wang et al. 2024a). While culturomics may not be effective and sufficient in quantifying species abundance, it is however the most suitable approach to obtain a comprehensive and viable repertoire of the microbiota (Dickson 2017; Togo et al. 2019a). The use of culturomics techniques has successfully led to the isolation and identification of a large repertoire of previously undetected and unculturable bacteria from the gut (Lagier et al. 2016, 2018; Cheema et al. 2023).

Previous studies have optimized a variety of rapid, economical, and effective culturomics techniques for the isolation of different types of bacteria from the gut microbiota of both humans and animals (Lagier et al. 2012, 2015; Chang et al. 2019; Hou et al. 2019, 2023; Wang et al. 2021b; Wan et al. 2023; Huang et al. 2023). Unlike the gut microbiota, there is yet a widely recognized robust culturomics technique designed specifically for milk microbiota. Recently, Wang et al. (2024a) successfully characterized the breast milk microbiota using a viable and effective culturomics strategy. Their study provided a solid foundation for the future application of the culturomics approach in milk microbiota research. Using four different culture media, conditions, and MALDI-TOF MS analysis, they identified 6601 colonies and obtained 865 bacterial strains, representing 54 species, 21 genera, and 4 phyla. Furthermore, they reportedly cultivated over 94.4% of the total bacterial species present in the milk samples with high diversity and a 57.0% reduction of workload (Wang et al. 2024a). Previously also, Togo and colleagues (Togo et al. 2018, 2019b) developed culturomics techniques for characterizing human milk microbiota from healthy breastfeeding African women. These separate studies isolated novel bacterial species, including *Anaerolactibacter massiliensis*, *Acidipropionibacterium timonense, Lactomassilus timonensis*, *Lactimicrobium massiliense*, and *Galactobacillus timonensis* using culture-based culturomics approach. These few instances where culturomics was applied in milk microbiota (Togo et al. 2018, 2019b; Wang et al. 2024a) improved our understanding of the robustness of culture-dependent techniques in characterizing viable microbial community, establishing, and preserving a comprehensive repertoire of bacteria in milk. As current knowledge of microbiota continues to increase, traditional culture-dependent methods may proportionally evolve to provide cost-effective strategies for identifying novel microorganisms and understanding the microbiota.

### Culture-independent omics approaches

The development of culture-independent high throughput sequencing (‘omics’) technologies has revealed the ecological intricacies of microbial communities across a wide range of environments, including milk. These technologies allow biologists to study the true diversity of the bacterial world thus, revolutionizing the fields of microbiology and microbial ecology. Since culture-dependent techniques only reveal culturable bacteria which represent a fraction of bacterial communities in a niche, culture-independent approaches use DNA, RNA, proteins, and metabolites to characterize the microbiota (Ruiz et al. 2019b; Chakraborty et al. 2023; Naqvi et al. 2024). Despite experimental biases and other limitations, the culture-independent techniques can detect previously unknown or yet-to-be-cultured bacterial groups with high sample throughput regardless of microbial viability. Additionally, omics technologies have been used to identify the relationships between microbial composition or structure with functions (Couvillion et al. 2023). A wide range of culture-independent omics approaches for milk microbiota profiling are available and have been increasingly used in the last two decades.

### Sequencing-based omics techniques

#### 16S and shotgun metagenomics

The 16S rRNA gene amplicon sequencing is the most commonly used culture-independent approach for milk microbiota profiling. While this approach provides comprehensive compositional, structural, and taxonomic information, shotgun metagenomics provides deeper insight into fine-resolution taxonomic diversity (at species, subspecies, and strain levels) and functional features in microbial communities by analyzing gene sequences that encode for functional RNAs or proteins (Moossavi et al. 2019a; Couvillion et al. 2023; Sun et al. 2024). Over the years, several studies have characterized the milk microbiota from both healthy and diseased (mastitis) hosts (e.g. humans, cows, goats, etc.) using metagenomics (Kordy et al. 2020; Olshan et al. 2021; Polveiro et al. 2022; Dahlberg et al. 2023; Khasapane et al. 2023; Alessandri et al. 2023a; Burakova et al. 2023; Ajeeb et al. 2024). Recent studies used metagenomic sequencing to characterize and compare the milk microbiota from cows with mastitis and healthy controls with some studies annotating the metagenomic sequences to identify and relate the microbiota with functional genes and metabolic pathways (Hoque et al. 2020; Tarrah et al. 2022; Alessandri et al. 2023b; Khasapane et al. 2023; Sahoo et al. 2024; Zhang et al. 2024a, b; Ran et al. 2024; Sabarish and Dhanasekaran 2024; Salman et al. 2024). Similarly, metagenomic analysis has been used in different studies to characterize the microbiota of human breast milk from women with and without mastitis (Jiménez et al. 2015; Boix-Amorós et al. 2020; Hoque et al. 2020; Asbury et al. 2020; Castro et al. 2022; Ito et al. 2023; Ong et al. 2023; Sindi et al. 2023; Chen et al. 2023b; Filatava et al. 2023; Treven et al. 2024; Ran et al. 2024; Endika et al. 2024). In addition to microbial composition, structure, and diversity identified by metagenomics, other genomic determinants, including immunologic profiles, metabolic, virulence, and antibiotic resistance determinants have been associated with microbiota perturbations and mastitis in women (Castro et al. 2022; Ito et al. 2023; Ong et al. 2023; Sindi et al. 2023; Treven et al. 2024; Ran et al. 2024; Endika et al. 2024).

The characterization of the microbiota using sequenced-based approaches involves the profiling of the whole set of microbial genomes within the community or target sequencing of the 16S RNA gene. The latter combines the amplification and sequencing of a fragment of the 16S rRNA gene to characterize the microbiota. Being the most conserved and targeted gene in bacteria, the 16S rRNA gene carries hypervariable regions (V1–V9) that bind to a pair of primers and then get amplified to capture taxonomic information (Addis et al. 2016; Sarangi et al. 2019; Parente et al. 2020; Lopez Leyva et al. 2021). While the 16S rRNA remains the most common approach in characterizing the milk microbiota, some limitations, including primer specificity, low bacterial load in milk, varied experimental platforms and procedures, variability in diversity estimates, loss of diversity due to amplification biases, etc. have been recognized (Jumpstart Consortium Human Microbiome Project Data Generation Working Group 2012; Logares et al. 2014; Fitzstevens et al. 2017; Sarangi et al. 2019; Lopez Leyva et al. 2021). These contribute to the inconsistent reports on prevalent or core genera or species associated with milk from healthy or mastitis-suffering hosts. Alternatively, shotgun metagenomics aims at sequencing DNA directly from available genomic material in a sample and then assembles contiguous sequences or entire genomes in order to assign high-resolution functional and taxonomic information (Sarangi et al. 2019; Almeida et al. 2019; Peterson et al. 2021; Usyk et al. 2023). Unlike the 16S rRNA gene sequencing, this approach does not target or amplify a specific gene. Therefore, it overcomes the bias associated with gene amplification and is generally regarded as the gold standard for microbiome characterization (Lopez Leyva et al. 2021). Large amounts of reads that are complex and difficult to assemble de novo are often generated and usually mapped and annotated for quantitative analysis using different bioinformatics pipelines and databases for high-resolution taxonomic and functional characterization of metagenomes in microbial communities. Metagenomic sequencing approaches are DNA-based techniques that describe the presence of microorganisms and genes within the community but are incapable of characterizing transcriptional profiles of the entire microbial community or individual microorganisms in the community (Couvillion et al. 2023; Cheema et al. 2023).

#### Metatranscriptomics

The previously discussed sequenced-based metagenomics approaches describe the structure and composition of microbes and genes within a community but not the functional activity of individual organisms or the whole community. Metatranscriptomics characterizes the transcriptional profiles of microbial communities, and it therefore provides insight into the active functional profile of the microbiome (Aguiar-Pulido et al. 2016; An et al. 2019; Arıkan and Muth 2023). Under specific conditions, metatranscriptomics analysis examines the total RNA within the community (metatranscriptome), which provides useful information on gene expression within the community over time. By capturing the total mRNA in a sample under specific conditions, the metatranscriptome provides information on the gene expression within the community at a specific time. Using metatranscriptomics, the pool of RNA transcript expressed in a community at a given time is analyzed, thus simultaneously allowing the characterization of both microbial abundance (rRNA) and gene expression (mRNA) in a community (Tveit et al. 2014; Addis et al. 2016; Zhang et al. 2021a, b). Metatranscriptomics was initially applied using hybridization or qPCR-based techniques (Higuchi et al. 1993; Simon and Daniel 2011). However, with the advancement in sequencing technologies, RNA-Seq has been established as the gold standard mainly due to the lack of reference isolates and the high diversity of microbial communities (Zhang et al. 2021a, b; Arıkan and Muth 2023).

Metatranscriptomics has been used in both humans and animals to characterize the temporal gene expression and functional analysis in mammary cells and milk during the lactation cycle (Martin Carli et al. 2020; Twigger et al. 2022; Wu et al. 2022; Xuan et al. 2022; Smilowitz et al. 2023; LeMaster et al. 2023; Xia et al. 2023; Doerfler et al. 2024; Zorc et al. 2024; Pozovnikova et al. 2024). Milk metatranscriptomics studies have primarily centered on examining host RNA in milk for information regarding host cell function and health (e.g. somatic cells) rather than specific or overall community microbial functions (Couvillion et al. 2023). In a recent study integrating milk metagenomics and metatranscriptomics, Zhang et al. (2024a, b) demonstrated the association between elevated somatic cell counts and high relative abundance of *Sphingomonas* and *Ralstonia* in cows with subclinical mastitis. The expression of bovine uridine phosphorylase 1 and transcobalamin 1 positively correlated with the relative abundance of *Sphingomonas* and *Ralstonia*. Their study further revealed distinct functional alternations in some microbial processes. Another obstacle to limited microbial metatranscriptomics study is the difficulty in differentiating between microbial and host RNA in milk. As earlier stated, microbial biomass in milk is low and could easily be dominated by the more abundant host RNA (Couvillion et al. 2023). In addition to providing active functional information, total RNA metatranscriptomics could also provide compositional and taxonomic insights into microbial communities (Xue et al. 2020; Hempel et al. 2023; Thøgersen et al. 2024). Integrating metagenomic data can facilitate metatranscriptomics analyses and assembly (Wu et al. 2023a; Hempel et al. 2023; Zhang et al. 2024a, b). Simultaneous profiling of microbial and host transcriptome to characterize microbial and host responses in disease has been reported (Pérez-Losada et al. 2015; Castro-Nallar et al. 2015; Ramos-Tapia et al. 2023). Therefore, this dual and integrated approach could be applied in milk to understand functional and taxonomic interactions between microbes and hosts during mastitis.

## Mass spectrometry-based omics techniques: metaproteomics, metametabolomics, and lipidomics

Beyond structural and taxonomic profiling, it is important to evaluate the functional roles and phenotypic features driving microbial communities as well as their impact on the host. The use of mass spectrometry-based omics approaches such as metaproteomics, metametabolomics, and lipidomics to characterize microbial communities has facilitated our understanding of the functional roles of microorganisms within their communities. Metaproteomics characterizes the entire protein content of microbiota and thus provides a direct measure of the functionality of the microbiota (Lamendella et al. 2012; Arıkan and Muth 2023). Metaproteomics also enables the evaluation and identification of splicing variants, post-translational modifications, protein complexes, and protein–protein interactions of microbial communities across ecosystems (Ahrens et al. 2010; Addis et al. 2016). The significant technological advances in recent decades now allow the increasing use of the metaproteomics approach in microbiome research, partly due to its affordability, feasibility, optimized workflows, and the application of advanced metaproteomics and computational data analysis tools and pipelines (Schiebenhoefer et al. 2019; Kleiner 2019; Sajulga et al. 2020; Van Den Bossche et al. 2021b; Arıkan and Muth 2023; Zhu et al. 2024b; Petrone et al. 2024). Currently, metaproteomics is primarily used to characterize the structure of the microbiota based on protein biomass, microbial interactions, and substrate utilization by individual microbes as well as the overall community metabolism and physiology (Kleiner 2019; Zhao et al. 2022; Buthasane et al. 2023; Chen et al. 2024; Shi et al. 2024a, b).

In recent years, several proteomics studies characterized the functional profiles and changes in milk peptides and proteins from healthy and mastitis-suffering hosts (Thomas et al. 2016; Tanamati et al. 2020; Bathla et al. 2020; Turk et al. 2021; Winther et al. 2023; Rešetar Maslov et al. 2023; Vanzin et al. 2023; O’Reilly et al. 2024). Metaproteomics profiling has been previously used to demonstrate proteins associated with antimicrobial resistance in raw bovine milk (Piras et al. 2020). Similarly, metaproteomics analysis was used to unravel the functional changes in the gastrointestinal tract microbiome of colorectal cancer patients and the associated taxonomic perturbations in gut bacteria (Long et al. 2020). The integration of metaproteomics in microbiome research bridges metagenomics and metatranscriptomics information to both phenotypic and metabolic information available in the metabolome (Van Den Bossche et al. 2021a). Through metaproteomics characterization, functional profiling, differential abundance, and taxonomic analysis can be conducted at the protein or peptide level (Zhang and Figeys 2019). Metaproteome evaluation involves a dynamic and broad range peptide identification with high sensitivity hence, requiring the state-of-the-art approach such as coupling extensive liquid chromatography (LC) separation systems with high-resolution mass spectrometers (MS) (Verberkmoes et al. 2009; Arıkan and Muth 2023). However, the major challenges in the application of metaproteomics in milk microbiome research are the high diversity and broad range of protein abundance, the presence of host (contaminating) proteins in milk, standardization and reproducibility of experimental protocols, and the unavailability of optimized bioinformatics pipelines and databases for the annotation and construction sequences for peptide identification of many community members (Kolmeder and de Vos 2014; Tanca et al. 2014; Kunath et al. 2019; Zhang and Figeys 2019; Van Den Bossche et al. 2021b; Blakeley-Ruiz and Kleiner 2022). Interestingly, integrating metagenomics data from the milk sample being studied is significant for providing insights for efficient annotation and identification of proteins (Tanca et al. 2014; Gouveia et al. 2020; Marzano et al. 2023).

Metametabolomics systematically identifies and quantifies all metabolites (usually small molecules with molecular weights less than 2000 Da) produced by microbial communities (Grim et al. 2019; McAtamney et al. 2023). Metametabolomics not only provides overall information on the physiological state of the microbiome but also the signaling processes, pathway regulations, and phenotypes (Arıkan and Muth 2023). The metabolome of a microbial ecosystem is often regarded as the most direct indicator of the health (eubiosis) or dysbiosis (perturbations of homeostasis) of an ecosystem (Bernini et al. 2009). Metametabolomics evaluation of the microbiome also provides useful information about microbial interactions within the community, metabolite biomarkers, novel enzymes as well as the host environment (Bauermeister et al. 2022). The presence of certain environmental factors such as diet, environmental stressors, and xenobiotics may directly impact metabolomic profiling (Manor et al. 2014; Aguiar-Pulido et al. 2016). Metametabolomics has been broadly and increasingly applied in different areas including translational microbiome research. Different sizes and types of metabolites can be introduced to milk primarily as a result of microbial metabolism or from maternal origins through mammary epithelial cell secretions, serum, or somatic cell activity (Suh 2022; Ali et al. 2022; Stinson and George 2023; Hailemariam et al. 2023). Also, the overall quality and safety of milk, especially organoleptic properties, nutritional value, coagulation activity, heat, and storage stability can be influenced by the presence of certain metabolites (Couvillion et al. 2023; Hailemariam et al. 2023).

While the majority of milk metabolomics studies aimed at unraveling the nutritional value of milk across animal species, humans, lactational stages, geography, or infant formula (Qian et al. 2016; Bardanzellu et al. 2019; Poulsen et al. 2022; Pintus et al. 2023; Su et al. 2023; Lemas et al. 2023; Yan et al. 2024), fewer (and recent) investigations are now analyzing the metametabolome of mastitis milk of both animal and human origins (Dervishi et al. 2017; Xi et al. 2017; Zhang et al. 2022a; Miyata et al. 2024; Wang et al. 2024d). Recent studies of milk metametabolomes reported higher amino and organic acids in milk from hosts suffering from mastitis (Zhu et al. 2023, 2024a; Miyata et al. 2024). Furthermore, pathway analysis demonstrated amino acids metabolism and energy metabolism as the major mechanisms of alterations in milk metabolomes during mastitis (Zhu et al. 2023, 2024a). Zhu et al. (2021) and Xi et al. (2017) similarly reported an association between mastitis and alterations in the biosynthesis of tryptophan, tyrosine, and phenylalanine in the tricarboxylic acid cycle as well as downregulation of energy, carbohydrate, and lipid. In their study, Gómez-Gallego et al. (2018) found a correlation between the relative abundance of breast milk bacteria and specific milk metabolites from breast milk obtained from 79 healthy lactating women from Spain, South Africa, Finland, and China. This suggests potential functional interactions between milk microbiota and metabolites. Microbial metabolomes are commonly characterized using either mass spectrometry (MS) (coupled with gas chromatography (GC), capillary electrophoresis (CE), liquid chromatography (LC)) or nuclear magnetic resonance (NMR) spectroscopy. NMR is economical, non-destructive, and has the advantage of direct detection and quantification of metabolites, however, it is relatively less sensitive with low throughput (Emwas et al. 2019; Arıkan and Muth 2023; Zniber et al. 2024). On the other hand, MS (coupled with LC or GS) is highly sensitivity and capable of detecting a wider range of molecules, however, it is a destructive technique and suffers technical challenges in quantification of molecules (Abram 2015; Arıkan and Muth 2023). The common limitations associated with the use of metabolomics in microbiome research are the non-uniformity in sample preparation and molecules profiling, the prevalence of unknown molecules in untargeted analysis, difficulties in annotation and bioinformatics analysis, and lack of unified databases (Alseekh et al. 2021; Cai et al. 2022; Niranjan et al. 2023).

While the use of Mass spectrometry (MS) (especially MALDI MS) in disease diagnosis and biomarker profiling has yielded significant results over the years, it is however limited in some ways. MS techniques rely on complex and laborious upfront sample preparation and separation, sample quality/homogeneity, as well as time-consuming analysis of each sample by the mass analyzer (Piras et al. 2022; Karch et al. 2022). Additionally, the upfront sample preparation often results in the loss of high-resolution proteoform-related information due to enzymatic digestion (Schaffer et al. 2019; Melby et al. 2021; Kaulich et al. 2024). Ion mobility and dissociation efficiency of protein complexes also constitute additional challenges of mass spectrometric analysis (Marklund and Benesch 2019; Karch et al. 2022). To overcome the limitations associated with MS, liquid atmospheric pressure (LAP)-MALDI MS technique has been developed for high throughput mass spectrometric analysis of biomolecules for both disease diagnosis and phenotypic profiling of microbial communities (Hale et al. 2018, 2019; Piras et al. 2022; Lellman et al. 2023; Challen et al. 2024). The analytical advances and new functionalities of LAP-MALDI MS use atmospheric pressure to analyze liquid samples (now easily prepared) which are introduced to analyzers with less matrix-cluster ions interference (Ryumin and Cramer 2018; Piras et al. 2022; Challen et al. 2023). In contrast to the conventional MALDI MS, LAP-MALDI allows stable ion yields, homogenous samples (liquid droplets), and record-breaking sample analysis speed (~ 60 samples per second). Additionally, LAP-MALDI is coupled with a heated inlet capillary that predominantly produces electrospray ionization (ESI)‐like multiply charged biomolecules (e.g., proteins and peptides) capable of detecting a limited *m*/*z* range (Hale et al. 2019; Krenkel et al. 2022; Challen et al. 2023, 2024).

In recent years, LAP-MALDI MS has been increasingly utilized for early and rapid detection of pre-clinical mastitis and the diagnosis of diseases, including bovine tuberculosis with high specificity and sensitivity (Hale et al. 2019; Piras et al. 2022; Krenkel et al. 2022; Challen et al. 2023; Lellman et al. 2023). To analyze milk microbial and/or host-associated biomarkers and AMR determinants in response to mastitis, only small amounts of milk are required. These small volumes of milk are prepared using a rapid preparation protocol that includes a one-pot/two-step method that allows the detection of proteins, peptides, and lipids within the mass spectral profile, hence rapidly detecting mastitis usually days before the onset of clinical manifestations (Hale et al. 2019; Piras et al. 2022). LAP-MALDI is designed to work with high-throughput mass spectrometers to simultaneously detect a wide range of heterogeneous metabolites and biomolecules, including proteins, peptides, and lipids (Piras et al. 2022; Challen et al. 2023). In a study involving 135 milk samples from 109 cows, a high abundance of many multiply charged ions in the *m*/*z* range of 600–1000 and attributable to peptides were detected in mastitis samples (Hale et al. 2019). Deconvolution of the mass spectra indicated multiply charged ions of *m*/*z* 600 and above in mastitis milk samples (62/135). LAP-MALDI MS was also used in another study to detect clinical and pre-clinical bovine mastitis from approximately 12,000 milk samples obtained from 500 cows within 6 months. LAP-MALDI MS profiles showed the presence of lipids (with the most abundant ions originating from triacylglycerols, phosphocholines, diacylglycerols, and sphingomyelins) and proteins/protein fragments as multiply charged ion species acquired over the m/z range of 100–2000 (Piras et al. 2022). Furthermore, a high abundance of isracidin-containing peptide ion, β-casein, and α-s_1_-casein fragments was identified in mastitis milk as earlier reported (Hale et al. 2019). The high sensitivity and specificity of LAP-MALDI MS allow rapid detection of bovine mastitis two days before the onset of clinical signs and symptoms (Piras et al. 2022).

In addition to the detection of bovine mastitis, LAP-MALDI MS has been used to detect antimicrobial resistance (AMR) determinants in mastitis milk. Using a similar protocol and experimental setup for mastitis detection, LAP-MALDI MS can effectively and simultaneously detect beta-lactamase-based AMR from milk even faster and simpler than mastitis, obtaining the required mass spectral data within a few seconds (Piras et al. 2022). Unlike conventional AMR testing protocol, the advances in LAP-MALDI MS significantly reduce the overall time to detect AMR effectively.

Lipids are another major component of mammalian milk that play vital biological roles. The milk lipidome contains complex and strategically packaged fat globules that encapsulate triacylglycerides within its core while the outer membrane is made of phospholipids and cholesterol (George et al. 2020). Milk lipidomics analysis characterizes and quantifies the structure and function of intact lipid molecules in milk (Yue et al. 2021; Liu and Rochfort 2023). The increasing use of lipidomics profiling in microbiome research in recent years has not only revealed the interplay between the microbiota and lipids but also provides valuable insights into disease diagnosis (Hornburg et al. 2023; Walczak-Skierska et al. 2023; Pan et al. 2024a; Thangaraj et al. 2024; Li et al. 2024a, b). Recent reports of milk lipids profiling of several mammalian species have provided a comprehensive dataset that describes 3454 triacylglycerides and 514 polar lipid molecules (Liu et al. 2020a, b; Manis et al. 2021; Zhang et al. 2022b; Zhao et al. 2022; Gao et al. 2022; Sun et al. 2022; Wu et al. 2023b; Pan et al. 2024a).

Milk lipidomics analysis has provided useful insights into several bioactive lipids-mediated inflammatory responses and changes in lipid contents associated with mastitis and milk microbiota dysbiosis (Ceciliani et al. 2020, 2021; Couvillion et al. 2023; Luo et al. 2023). Notable changes in milk lipidome, lipids metabolism, and eventual decrease in milk quality have been observed in milk from mastitis-suffering hosts (Ceciliani et al. 2020, 2021, 2022; Ganeshalingam et al. 2022; George et al. 2023; Luo et al. 2023; Pan et al. 2024b; Hyötyläinen et al. 2024). For instance, Ceciliani et al. (2021) reported significant changes in major lipid groups including sphingomyelins and triacylglycerols in milk from cows with non-*aureus* staphylococci associated subclinical intramammary infection. Similarly, Luo et al. (2023) showed a correlation between the changes in mastitis milk lipid content and the metabolisms of glycerol phospholipid, arachidonic acid, and α -linolenic acid, thus, describing the role of mastitis in triggering abnormal lipid metabolism as well as driving milk microbiota diversity. This further highlights the milk lipidome as a potential biomarker for mastitis diagnosis and milk safety assessment. The rapid innovations and diversification in lipidomics methods (e.g. mass spectrometry techniques) have facilitated the continuous improvement of milk lipidomics, unraveling previously unknown lipid-microbiota interactions in milk ecosystems. The current analytical techniques employed for lipidomics analysis include LC–MS, CE-MS, ESI–MS, MALDI-MS, GC–MS, NMR, and shotgun lipidomics (George et al. 2020; Yue et al. 2021; Thangaraj et al. 2024; Li et al. 2024a, b). While there are significant advances in milk lipidomics, there are yet some limitations with this mass spectrometry-based approach. Unfortunately, the unavailability of a potent lipid separation system capable of differentiating phospholipids from triacylglycerols and the lack of conventional standards for absolute lipid quantification at the species level are major limitations to the use of lipidomics in milk microbiome research (Liu et al. 2018; Yue et al. 2021; Liu and Rochfort 2023).

Milk microbiota across humans and many animal species are now increasingly shown to harbor resistome-related proteins which have a significant effect on mastitis and host health (Piras et al. 2020, 2024; Warder et al. 2021; Qin et al. 2023; Holman et al. 2023; Rahmeh et al. 2024). The genes of the resistome in the milk microbiota code for different proteins associated with antimicrobial resistance. These genes are easily acquired and/or exchanged among the members of the microbiota, and their expressions are often induced by antibiotic use or misuse (Baquero et al. 2019; Hwengwere et al. 2022). Bottom-up proteomics has been used to identify and characterize resistome proteins in milk across different animal species (Piras et al. 2020, 2024). Understanding the comprehensive structure of the milk microbiome, including the community (and microbial) protein expression (e.g., resistome proteins) could unravel the full functionality of the community and the pathophysiology of mastitis. The use of bottom-up proteomics and metaproteomics profiling of biological fluids, including milk allows high throughput identification of different proteins, protein fragments, and proteoforms from different microorganisms in a niche (Zhang et al. 2019). Regardless of their phylogenetic lineages, all host and microbial-associated proteins mediating host-microbiome interactions in health or disease can be identified and quantified (Zhang et al. 2018, 2024a, b; Levi Mortera et al. 2024). In a recent study, a bottom-up proteomics approach unraveled a high abundance of resistome proteins in Podolica cow milk (Piras et al. 2024). AMR-specific proteins identified in the study included only proteins associated with tetracycline resistance and beta-lactamases. The presence of AMR-specific proteins in milk could originate from bacteria from the environment, teat of the udder, or within the mammary gland (Piras et al. 2024). Similarly, Piras et al. (2020) previously identified 29 AMR-specific proteins/proteoforms, including β-lactamase and Aminoglycoside N(6′)-acetyltransferase in milk samples. The presence of these resistome proteins demonstrate active metabolic activities by microbes expressing these proteins within the milk microbiome. The presence of these specific AMR proteins could provide vital information about the composition, diversity, and structure of the microbial community and associated biomolecules. While metaproteomics provides detailed taxonomical and functional properties of the microbiome, bottom-up proteomics holds great promise in unraveling the distribution of microbial and host-associated proteins that could drive the community structure and functions as well as mastitis pathophysiology.

## Milk-omics approaches and data integration: multi-omics and functional implications

The simultaneous systems-based application of milk-omics approaches (multi-omics) to study milk microbiome is expected to generate new knowledge and open new avenues for a broader, finer, and holistic structural and functional understanding of the milk ecosystem in relation to health and disease (e.g. mastitis). The milk multi-omics approach combines multiple and heterogeneous independent data sets that could arrive at similar and interlinked conclusions with higher confidence supporting specific hypotheses. Each omics approach provides a specific and unique phenotypic and functional perspective of the community. However, by integrating these approaches and datasets through multi-omics, the overall community interactions, dynamics, functionalities, and patterns are unraveled in an unprecedented manner (Chetty and Blekhman 2024; Jiang et al. 2024; De Paepe et al. 2024). While single omics studies have undoubtedly contributed to our current understanding of mastitis, they often provide limited information about mastitis, targeting only a single biological viewpoint which is insufficient to provide system-wide information that is necessary for elucidating the biological footprints and molecular mechanisms driving mastitis pathogenesis (Subramanian et al. 2020; Wang et al. 2024c). Additionally, integrating multi-omics approaches and multi-dimensional datasets could unravel novel and previously unknown spatiotemporal microbial community relationships and/or interactions, thereby providing a complete, detailed, and multi-layer view of the ecosystem (Xu et al. 2024a; Wang et al. 2024c).

The multi-omics approach comprising metagenomics, transcriptomics, proteomics, metabolomics, and lipidomics in addition to culturomics has in recent years been applied in medicine, human diseases, environmental science, and agriculture to study complex ecosystems (e.g. gut) and develop sustainable diagnostic and treatment regimes (Chung and Kang 2019; Liu et al. 2022; Snajder et al. 2023; Tilgam et al. 2024; Ren et al. 2024). However, this approach is still growing and yet to be extensively applied in studying mastitis and raw milk microbiome. Recently, some studies have integrated a multi-omics approach to study mastitis at a systemic level (Xu et al. 2024a; Wang et al. 2024b; De Paepe et al. 2024; Zhang et al. 2024a, b). These studies revealed novel insights into the biological and molecular signatures of mastitis thus highlighting the power of the multi-omics approach in deepening our understanding of integrating the milk microbiome signatures in mastitis. Recently, Wang et al. (2024c) applied a multi-omics approach to study the [epi]genomic signatures and regulatory mechanisms in mastitis by integrating whole genome-wide DNA methylation sequencing data (WGMS), small RNA sequencing data (miRNA), and RNA sequencing data (mRNA and lncRNA) from bovine milk. Their study improved current understanding and identified detailed biological signatures and genetic mechanisms underlying mastitis. Similarly, a combined milk microbiota and metabolomics study (using amplicon sequencing and nuclear magnetic resonance spectroscopy techniques) revealed a strong disturbance of the microbiota (lower diversity and richness) with an altered energy and amino acids metabolism in milk from mastitis host (Zhu et al. 2024a). Correlations between milk microbiota composition, metabolites, and transcriptional profiles showed potential relationships between microbial genera, metabolite biomarkers, and transcriptional patterns of some genes in mastitis milk (Bellassi et al. 2021; Zhang et al. 2024a, b). Bellasi and colleagues (Bellassi et al. 2021) used a combined metagenomics and metabolomics approach to provide a comprehensive milk-omics landscape of raw cow milk. Their findings showed a significant correlation between the metagenomic profile and some milk metabolites, with *Dermabacteraceae, Pseudomonadaceae*, and *Staphylococcaceae* having direct and stronger correlations with those discriminant metabolites. The extensive application of multi-omics approach and data integration in gut microbiome research has shown stability in metabolome and proteome profiles despite perturbations in microbial composition, interestingly depicting functional redundancy that could not have been deciphered using an individual omics approach (Gierse et al. 2020; Muller et al. 2024). An integrated multi-omics approach comprising metagenomics, metaproteomics, metatranscriptomics, and metabolomics was used to link microbial community composition and structure to functional signatures and metabolic biomarkers in faecal samples of individuals with type 1 diabetes mellitus and colon cancer (Heintz-Buschart et al. 2016; Kunath et al. 2022; Busi et al. 2023; Bai et al. 2024). Similarly, integrating a multi-omics approach in milk microbiome research could generate new knowledge, improve the current understanding of the functional and structural signatures in the milk ecosystem, and provide useful insights on mastitis development and mitigating strategies.

Current microbiome research integrating the multi-omics approach has the potential of unraveling and attributing deeper functional changes in the overall community biomarkers and gene expression to specific community members or taxa over time and space. Integrating metagenomics, metatranscriptomics, metabolomics, and lipidomics in a system-based multi-omics approach would provide a clearer perspective of the community from genes to phenotype (Aguiar-Pulido et al. 2016). Despite the huge benefits of the multi-omics approach in milk microbiome research, some challenges persist in its application. Integrating meta-omics data requires significant financial and computational resources and high-level cross-disciplinary expertise. Conducting experimental studies using multiple platforms, techniques, and protocols tends to produce heterogeneous and disparate data sets with varying missing values, reproducibility issues, data availability, scalability and performance of tools, and difficulties in making biological inferences (Couvillion et al. 2023; Arıkan and Muth 2023). Multi-omics data generated across multiple platforms of analysis would require efficient integration methods strong enough to overcome differences in resolution and technique-associated biases to draw meaningful biological interpretations.

The advances and application of omics technologies have increased the current understanding of the functionality of microbial communities and their impact on health and disease. While single omics studies have undoubtedly contributed to our understanding of milk microbiome and mastitis, they often provide limited information, targeting only a single biological viewpoint which is insufficient to provide system-wide information that is necessary for elucidating the biological footprints and molecular mechanisms driving mastitis pathogenesis. Integrating a multi-omics approach in milk microbiome research could generate new knowledge and improve the current understanding of the structural and functional signatures in the milk ecosystem and also provide insights for the development of mastitis control strategies.

Beyond the mechanistic and compositional profiling of the milk microbiome, the use of a multi-omics approach would unravel deeper ecological and functional intricacies in milk which could be used as a model system. Specific milk microbe-microbe and microbe-environment interactions in health and disease across time and space (during lactation) could be explored using a multi-omics approach in future research. This may provide unprecedented insights into the network of interactions between the milk microbiota and the proteome, transcriptome, and metabolome in health and during the cause of mastitis. The use of multi-omics approaches in microbiome research will in the future require the standardization of methods from sampling to experimental protocols and data generation, annotation, integration, and interpretation. The documentation of standardized protocols and workflows will enhance innovation in milk microbiome research.

## Application of omics in characterizing antimicrobial resistance in milk microbiome

The escalating menace of antimicrobial resistance (AMR) continues to constitute a significant global burden for public health. The abundance and high diversity of bacterial species in [mastitis] milk enhance the rapid accumulation and acquisition of multiple antimicrobial resistance genes, widespread horizontal gene transfer, and short generation times (Hoque et al. 2020; Zhang et al. 2021a, b; Tran and Dahlin 2024). Numerous studies have over the years characterized microbial structure and composition in mastitis and non-mastitis milk using different omics approaches. However, the overall resistome of the milk and the distribution of specific antibiotic resistance genes has been scarcely studied in milk microbiome. The dissemination of emergent AMR genes and pathogens by the milk could pose health risks to both humans and animals, thus facilitating different infections, including mastitis (Hoque et al. 2019; Rahmeh et al. 2024; Shi et al. 2024a, b; de Souza et al. 2024; Rahman et al. 2024). Mitigating the threat posed by AMR in the milk microbiome requires a deeper understanding of the molecular biomarkers and mechanisms driving AMR emergence and transfer as well as the integration of a multi-omics approach that will provide insight into the complex interactions in the microbiome.

In a recent study characterizing the microbial diversity and resistomes in mastitis and healthy cow milk in India’s coastal district of Odisha, a higher number of antimicrobial resistance genes were recorded in mastitis milk as compared to the non-mastitis healthy milk samples (Sahoo et al. 2024). This was reported in addition to the significantly higher bacterial abundance and diversity in mastitis milk. While a large pool of antimicrobial resistance genes against macrolides, tetracyclines, β-lactams, peptides, and fluoroquinolones were detected in mastitis milk, only a few antimicrobial resistance genes against β-lactams and aminoglycosides were identified in healthy milk samples, respectively (Sahoo et al. 2024). It is important to note that antimicrobial resistance genes are often ubiquitous in microbiomes, and they have been detected in most ([non]-pathogenic) bacteria within a niche (Tóth et al. 2020; Samarra et al. 2023; Sahoo et al. 2024; Cebeci 2024). A large-scale omics study of antibiotic resistomes in 2034 milk samples from California, United States showed significantly increased abundance and richness of antimicrobial resistance genes (Liu et al. 2020a, b). Specifically, 49 different antimicrobial resistance genes belonging to 15 antimicrobial resistance groups with 7 mechanisms of resistance were found in the milk samples. Most (80%) of the antimicrobial resistance genes were assigned to a bacterial host at the family level. The bacterial families harboring the predominant antimicrobial resistance genes were *Pseudomonadaceae*, *Enterobacteriaceae*, *Yersiniaceae*, and *Moraxellaceae* (Liu et al. 2020a, b).

In an attempt to provide insights into the role of the resistome in the severity of bovine clinical mastitis, Hoque et al. (2020) identified 2 unique groups consisting of 19 genes responsible for resistance to antibiotics and 11 genes for toxic metal resistance in the microbiome of mastitis milk. This report is in consonance with other findings reported elsewhere from mastitis milk of humans (Patel et al. 2017; Brinkac et al. 2017; Baron et al. 2018; Nhu and Young 2023), cows (Cheng et al. 2019; Hoque et al. 2019; Sharifi et al. 2023; Li et al. 2024a, b; Liu et al. 2024), and buffalo (Preethirani et al. 2015; Sun et al. 2021). These findings suggest that the milk microbiome of the mastitis host constitutes an important reservoir for the acquisition and distribution of AMR. An omics study of bovine colostrum microbiome resistome notably documented an increased diversity in the distribution of AMR genes in buffalo and cow colostrum microbiomes (Yasir et al. 2024). A total of 175 antimicrobial resistance genes and related variants were identified in cow and buffalo milk, with 55 genes occurring in both groups. The core resistome analysis detected AMR genes that confer resistance against multiple antibiotic classes including sulfonamide, tetracycline, fluoroquinolone, aminoglycoside, and peptide antibiotics (Yasir et al. 2024). Additionally, these AMR genes were associated with the most common antimicrobial resistance mechanisms including, antibiotic efflux, antibiotic inactivation, and antibiotic target alteration mechanisms.

Antimicrobial resistome profiling of milk microbiome with (or without) mastitis using a multi-omics approach may provide valuable insights into (1) identifying potential hotspots and reservoirs for the acquisition and distribution of AMR, (2) public health hazards associated with antimicrobial resistant infections and antibiotic use, (3) microbiome-scale knowledge of intrinsic resistance mechanisms, (4) improve therapeutic schemes optimization of antimicrobial use in the treatment and prevention of mastitis, and (5) developing a sustainable concerted One Health approach in mitigating the global menace of AMR.

## Data Availability

No datasets were generated or analysed during the current study.
